# Public perceptions of Lyme disease and climate change in southern Manitoba, Canada: making a case for strategic decoupling of climate and health messages

**DOI:** 10.1186/s12889-021-10614-1

**Published:** 2021-03-30

**Authors:** Laura Cameron, Rhéa Rocque, Kailey Penner, Ian Mauro

**Affiliations:** Prairie Climate Centre, University of Winnipeg, 515 Portage Ave, Winnipeg, Manitoba R3B 2E9 Canada

**Keywords:** Climate change, Lyme disease, Risk perception, Public perceptions, Canada

## Abstract

**Background:**

Despite scientific evidence that climate change has profound and far reaching implications for public health, translating this knowledge in a manner that supports citizen engagement, applied decision-making, and behavioural change can be challenging. This is especially true for complex vector-borne zoonotic diseases such as Lyme disease, a tick-borne disease which is increasing in range and impact across Canada and internationally in large part due to climate change. This exploratory research aims to better understand public risk perceptions of climate change and Lyme disease in order to increase engagement and motivate behavioural change.

**Methods:**

A focus group study involving 61 participants was conducted in three communities in the Canadian Prairie province of Manitoba in 2019. Focus groups were segmented by urban, rural, and urban-rural geographies, and between participants with high and low levels of self-reported concern regarding climate change.

**Results:**

Findings indicate a broad range of knowledge and risk perceptions on both climate change and Lyme disease, which seem to reflect the controversy and complexity of both issues in the larger public discourse. Participants in high climate concern groups were found to have greater climate change knowledge, higher perception of risk, and less skepticism than those in low concern groups. Participants outside of the urban centre were found to have more familiarity with ticks, Lyme disease, and preventative behaviours, identifying differential sources of resilience and vulnerability. Risk perceptions of climate change and Lyme disease were found to vary independently rather than correlate, meaning that high climate change risk perception did not necessarily indicate high Lyme disease risk perception and vice versa.

**Conclusions:**

This research contributes to the growing literature framing climate change as a public health issue, and suggests that in certain cases climate and health messages might be framed in a way that strategically decouples the issue when addressing climate skeptical audiences. A model showing the potential relationship between Lyme disease and climate change perceptions is proposed, and implications for engagement on climate change health impacts are discussed.

## Background

Climate change is now considered the “biggest global health threat of the 21st century” [1]. Climate health impacts vary across regions and demographics, ranging from heat-related illnesses, increased aeroallergens impacting asthma, worsened air pollution causing respiratory issues, mental health impacts of extreme weather events, threats to traditional and agricultural food security, and the spread of infectious diseases [2]. Indicative of the magnitude and multitude of threats that climate change poses to health globally, the scientific literature published on health and climate change more than tripled between 2007 and 2016 [3].

The swiftness and efficacy of our collective response to climate change – adaptation and mitigation to lessen the impacts – is critical to the resilience of populations around the world and the health outcomes societies will face [2]. Indeed, public health depends on the ability of citizens to engage with and understand risk, and risk perception studies are designed to gauge this ability, while risk communication research determines how to best engage with the public to support informed decision-making [4]. Understandings of public risk perception of climate change is essential, as it allows for proper design, implementation, and long-term support for policy and planning [5]. Risk perceptions can also serve as an important indicator of willingness to support action on climate change [6–9]. Even if adaptation plans exist, public support is unlikely until the risks of inaction have been successfully communicated [10].

It has been suggested that adopting public health frames to communicate climate change can maximize engagement from a wider audience [11–16]. Focusing on the health risks of climate change can make the issue more understandable, effective, and motivating by connecting it to personally relevant and relatable health issues such as allergies or heat stroke [16]. As a result, there is a growing body of research examining public health framing in climate communications, including several recent studies analysing this framing in news media in the US, New Zealand, and France [16–21]. To better understand the efficacy of health frames in climate communications, we must first understand public perceptions of the health risks associated with climate impacts.

While research on public awareness and perceptions of climate change health impacts has been undertaken around the world (e.g. [12, 22–25]), research in Canada is limited to date. In a review of the literature that does exist, Hathaway and Maibach found that relatively few North Americans associate climate change with health harms [24]. Other studies have similarly found that when prompted, Canadians can name some health impacts of climate change, but the overall link between health and climate change lacks salience for the public [26, 27]. Indeed, many people are not adopting preventative behaviours in response to existing public health and climate communication materials [28]. In light of this, researchers have called for more public health communication aimed at increasing the salience of climate change health impacts [26] and for “reframing climate change from an environmental to a public health issue” [27] (p. 11).

An emerging and significant climate-affected health outcome in the Canadian Prairie provinces is tick-borne Lyme disease. Lyme disease is caused by an infection of the bacteria *Borrelia burgdorferi*, transmitted to humans by *Ixodes scapularis* (commonly the blacklegged tick) in eastern and central North America and by *Ixodes pacificus* (the western blacklegged tick) in western Canada [29]. Blacklegged ticks are spreading northward, due in part to climate change and land use changes, bringing the risk of tick-borne Lyme disease to new regions in the US and Canada [30–34]. Lyme disease has now been reported in every province, from British Columbia to Prince Edward Island [30] and is the most commonly-reported vector-borne disease in the temperate world [35]. Future warming is expected to increase growth and reproductive rates of blacklegged ticks, as well as facilitating further range expansion, thereby increasing the risk of Lyme disease in new and endemic areas [31, 36] . These changing ecological factors interact with social-behavioural dimensions to shape variable Lyme disease risk across geographies [37].

In this light, Lyme disease is a significant focus for public health risk communication on climate change in Canada. While active and passive surveillance efforts are ongoing to monitor the environmental risk of Lyme disease in Manitoba and across Canada (e.g. [36, 38, 39]), experts looking to communicate the risks of emerging disease dynamics are seeking better understanding of public knowledge on Lyme disease. Understanding public risk perception can help target Lyme disease communications and drive the adoption of preventative behaviours [37, 40].

National studies in Canada have shown an increase in public awareness of Lyme disease risk following a 3 year national Lyme communication campaign led by the Public Health Agency of Canada [41], though a lower rate of increase in adoption of preventative behaviours towards tick bites [42]. Aenishaenslin et al. have shown that the impact of risk communication varies across regions in Canada, and communication strategies must be informed by an understanding of specific regional characteristics of risk to be more effective [41, 42].

As with the climate change health perceptions literature generally, there is a geographic gap in documented knowledge of Lyme disease risk perceptions in the province of Manitoba specifically. Only one known study has investigated risk perceptions of Lyme disease in Manitoba; through interviews with scientists, policy-makers, clinicians, and members of disease advocacy groups, Crang found a lack of knowledge among most Manitobans, and a desire for better communication on outcomes of research on Lyme disease [43].

This study responds to the need for a regional exploration of risk perceptions and communication in Manitoba, drawing from and contributing to the fields of climate change communication, health communication, risk perception, and psychology. Using a focus group methodology, across rural and urban communities in southern Manitoba, this research was structured around answering the following research questions:

1. What are the public perceptions of climate change and associated risks in Manitoba?
2. What are the public perceptions of Lyme disease and associated risks in Manitoba?
3. What is the relationship between public perception of both climate change and Lyme disease risks in Manitoba?

This study also seeks to understand the influence of visual communication materials on Lyme disease risk perception. Specific questions and responses regarding the efficacy of risk communication materials – in text, map, and video formats – will be the subject of a subsequent publication.

## Methods

### Study area and context

**Fig. 1:**
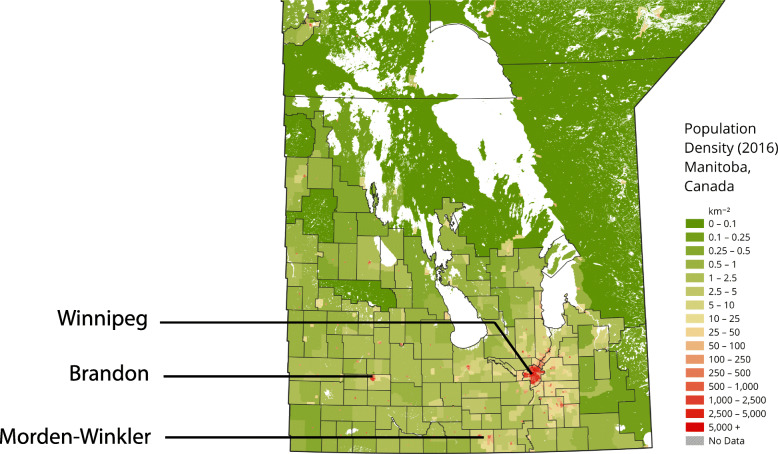
Map of study communities in southern Manitoba overlaid on population density of the province (base map from Wikimedia Creative Commons)

The study was conducted in southern Manitoba, a Prairie province in central Canada, across three communities representing a spectrum of urban to rural environments: Winnipeg (urban), Brandon (urban-rural), and Morden-Winkler and surrounding area (rural) (Fig. 1).

Although high-resolution public opinion data on climate change in Canada is limited, research has shown that discourse and perceptions of climate change in the Prairies are unique compared to other parts of Canada. At the national level, rural areas and Prairie provinces have a high level of climate skepticism and denial [44]. An estimated 42, 47, and 57% of people in Alberta, Saskatchewan, and Manitoba respectively, believe that the earth is warming partly or mostly because of human activity, compared to the national average of 60% [44]. Studies investigating climate opinions among specific groups in the Prairies, such as agricultural producers in Saskatchewan [45], have found similar results. In Manitoba, beliefs in anthropogenic climate change and the risk of its impacts are lower in rural areas than the national average. For instance, the percentage of people who believe that “earth is getting warmer partly or mostly because of human activity” in the federal ridings encompassing the study communities is 51% in Brandon-Souris (Brandon), 45% in Portage-Lisgar (Morden-Winkler), and 64% in Winnipeg (averaged across the eight ridings in the city), compared to the national average of 60%, from a regionally downscaled national survey (*n* > 9000) [44]. There is a need to better understand public risk perceptions surrounding climate change in this region to address climate skepticism and inform the specific needs of climate communications.

Lyme disease has emerged in Manitoba over the past two decades, as the blacklegged tick has moved north and west and become established in the province, in part due to climate change [46, 47]. Total reported cases of Lyme disease annually in Manitoba reported to the Public Health Agency of Canada have risen from 11 cases in 2009 to 81 in 2019 [48]. Blacklegged ticks live in wooded habitat, therefore people who spend time outdoors in or near wooded areas have a higher risk of coming into contact with Lyme-carrying ticks.

### Research design

An exploratory qualitative design based on the focus group method was used to better understand the public perceptions of climate change and Lyme disease [49, 50]. Focus groups allow a conversation to evolve which covers what the researcher wants to know, while allowing for elaborations and potentially unforeseen topics to arise [51]. Interactions between participants through conversation also provide greater insight into why opinions are held [52, 53], and allow a “process of joint sense-making to be studied in action” [54]. There are a variety of perspectives on the ideal size of a focus group [51]. While some researchers favour smaller groups to give more space to each voice, others suggest that larger groups can allow for richer dialogue between a range of perspectives [55, 56]. Peters suggests that a focus group should consist of six or more participants [50]. Herein, we aimed for ten participants per group, based on prior experience of the research team and the goal to capture a range of demographics and perspectives in each.

### Procedure

After obtaining ethics approval of the University of Winnipeg Human Research Ethics Board, the team collaborated with Probe Research to recruit participants and conduct the focus groups across the three communities. Probe Research is one of Manitoba’s main independent opinion research firms, with an extensive and representative list of Manitobans that was drawn upon to create a meaningful sample. With leadership from the research team, collaborators from Probe Research assisted with recruitment and focus group moderation, enabling an open discussion as an impartial third party [57]. Since the latter half of the focus groups tested materials developed by the research team, it was preferable to have an independent moderator to avoid any potential bias. Participants were recruited by phone and asked a short screening questionnaire to gather demographic and opinion information that was used to organize participants into groups. This questionnaire indicated that the study was about climate change but did not mention Lyme disease (participants were informed of the latter during the discussion at the beginning of the focus group as part of the informed consent process). Participants of a range of age, gender, cultural background, educational status, and time spent working/recreating outside were recruited. Anyone who worked for a media outlet, advertising company, in the field of climate science or at the University of Winnipeg, or anyone who had participated in a focus group within the past year, was excluded.

Two focus groups were held in each community, one with participants with “high” concern about climate change and one with “low” concern. Participants were segmented into groups according to their answers in a pre-screening process when asked to rate their level of concern regarding climate change on a scale from 1 to 4 (Table 1). Written individual informed consent was obtained by participants before starting the focus group discussions. All focus groups were conducted in November and December 2019 and were audio recorded and transcribed verbatim for analysis. Focus groups took place in community centres and offices and lasted on average 90 min. Participants received $100 in compensation for their time.

**Table 1:** Focus group demographics and climate opinions derived from the pre-screening questionnaire in the recruitment process. Climate opinions were rated on a scale from 1 (strongly disagree) to 4 (strongly agree) and median values and their interquartile ranges (IQR) are presented

				Climate opinions		Demographic information				
Group			Number of Participants	CC is human caused (median, IQR)	Level of CC concern (median, IQR)	Children at home (# yes)	Work outdoors (# yes)	Live/work on farm (# yes)	Education (# some or complete college/uni)	Age (range, average)
Winnipeg (urban)	W1	High concern	11	4 (3.5–4)	4 (4–4)	2	3	–	8	24–68, 49.3
	W2	Low concern	10	3 (3–3)	2 (2–3)	3	0	–	9	33–80, 56.3
Brandon (urban-rural)	B1	High concern	10	4 (4–4)	4 (4–4)	1	2	0	7	21–65, 48.2
	B2	Low concern	8	3 (3–3.25)	2 (1.75–3)	1	4	1	6	42–71, 59.6
Morden- Winkler (rural)	M1	High concern	11	4 (3–4)	4 (3–4)	4	3	1	9	33–70, 51.8
	M2	Low concern	11	3 (2.5–3)	2 (2–2)	5	2	2	5	25–68, 43.5

The focus group discussions were structured in three parts with open-ended questions and prompts regarding: 1) perspectives on climate change; 2) perspectives on Lyme disease; 3) relationship between climate change and Lyme disease perceptions; and 4) responses to three communications materials (video, map, and article) presented in randomized order (Table 2). Communications materials and facilitation questions were created in consultation with experts in the field and findings concerning the materials section (Part 4) are reported elsewhere. The questionnaire developed for this study is included in Appendix A.

**Table 2:** Structure of focus group discussions

	Discussion topics	Example discussion questions and prompts
Part 1	Climate change perceptions	*When I say the phrase ‘climate change’, what comes to mind?* *Have you seen any examples of changes in your community?*
Part 2	Lyme disease perceptions	*When I say the phrase ‘Lyme disease’, what comes to mind?* *When did you first hear about Lyme disease?*
Part 3	Relationship between climate change and Lyme disease perceptions	*Do you think there is a relationship between climate change and Lyme disease? Why or why not?*
Part 4	Responses to three communications materials (video, map, and article)	*When you review these various communication materials, what is most effective and communicating climate and Lyme risk? Why or why not?*

### Analyses

Inductive thematic analyses were conducted on the transcripts in a qualitative analysis software, NVivo 11.4. Analyses were overseen by four people on the research team. The qualitative analysis followed the approach outlined by Baxter [58]. Three rounds of coding were conducted independently by two researchers trained in qualitative methods, with comparison and discussion within each, and coding schemes were reviewed by the research team after each round. A coding structure of analytic thematic codes was iteratively developed [59]. Interpretation of the qualitative findings was guided in part by a frequency analysis of codes within and across groups. Frequency counts were understood to be an indicator of idea occurrence, but findings were not limited to this metric, considering frequency counts do not necessarily reflect the importance of the actual ideas being communicated. Indeed, in this analysis we wanted to better understand and access the perspectives climate skeptical audiences and associated unexpected findings, which are reasons to “avoid counting” [60]. Results from the analysis are reported below and the full coding scheme is included in Appendix B.

## Results

A wide range of knowledge and risk perceptions of both climate change and Lyme disease were found across focus groups, with some notable differences between geographies for Lyme disease perceptions and levels of climate concern for climate change perceptions. Results are shared following the focus group structure, beginning with public perceptions of climate change, and then followed by perceptions of Lyme disease. Findings suggest that risk perceptions of the two issues do not necessarily correlate; for instance, participants who believe that Lyme disease poses a high risk do not necessarily feel the same about climate change generally.

### Public perceptions of climate change

There was a wide range of knowledge and opinions on climate change among participants and groups. People who self-identified as having higher levels of concern about climate change often shared more factual information on the issue. In discussing climate change, many people drew on their own experiences, changes they have or have not witnessed, or what they have seen in the media. Regarding climate change perceptions, four major themes emerged during the analysis: causes; impacts; risk and awareness; and solutions and politics. Table 3 provides an overview of the major themes and sub-themes. Each theme is discussed, followed by notable differences between high and low concern groups. During analysis, it became clear that many codes were linked to an attitude of skepticism, which varied between high and low concern groups.

**Table 3:** Climate change perceptions themes and sub-themes that emerged through the process of qualitative analysis. The most common 5–6 sub-themes are shown per theme

Climate change perceptions			
Causes	Impacts	Risk and Awareness	Solutions and Politics
• Fossil fuels, emissions • Pollution • Urban sprawl, population growth • Manufactured problem • Responsibility for the problem	• Temperature changes • Weather changes and extremes • Health, human impacts • Ice, oceans, water • Pests, invasive species • Fire, drought	• Geographic risk • Temporal risk • Media coverage • Change is constant, natural • Skepticism or denial of the risk	• Adaptation • Individual, collective action • Disbelief or skepticism in solutions • Government and politics • Lack of scientific knowledge

#### Causes

Participants discussed the causes that they perceive to be driving or contributing to climate change. Several of the groups talked about air and water pollution, population growth, and urban sprawl as part of the causes of climate change. Several groups talked about a lack of trees in their communities. One person explained: “All I have to do is walk down to the river and watch all the garbage floating down … it’s a climate change thing when that’s going into the lake, and the lake is warming up …” . Later in that discussion someone else said: “I think people confuse climate change with just general pollution or poor waste management.” Other problems raised included reliance on fossil fuels, government silencing of scientists, and a culture of laziness and dependency impeding action on climate. People in several groups expressed a perception of climate change as a “manufactured problem” or as cyclical, normal, and/or natural, while some people said that humans are worsening the natural changes.

Something that came up often in discussions was the source and responsibility for the problem of climate change. People in four groups talked about responsibility lying in other places in the world that contribute greater amounts of emissions or pollution – larger cities like Toronto or larger countries like China or India – though there was some disagreement on this within two groups. Someone in Brandon explained their feeling that “because we’re smaller, there’s not that many of us polluting the country. Like for instance, smog in Toronto versus smog in Brandon, there’s not that many vehicles and everything else.” Global greenhouse gas emission increases were often misunderstood with more localized point source pollution issues.

#### Impacts

With regards to what participants understand to be climate impacts, the majority of groups talked about changing temperatures; many people said they believe temperatures are increasing, while a few people reported having experienced cooling in typically warm places. Most groups talked about flooding, citing recent flood experiences in southern Manitoba, and polar ice melting. Several groups brought up the unusually early snow storm and high precipitation in the region in the prior months, which caused the Red River Floodway around Winnipeg to be opened in the fall for the first time in history. Other common impacts noted included human health and livelihoods, sea level rise, reduced wildlife habitat, increasing wildfires and drought, decreased air quality, and new and invasive species. Health impacts noted included increase in asthma due to wildfire smoke and changing temperatures, migration and food shortages affecting nutrition, increasing diseases and introduction of new viruses. Three groups talked about the spread of insects and pests due to warming temperatures, one of which specifically named deer ticks but none mentioned Lyme disease. Overall, participants were seemingly more unified and better able to discuss observed climate impacts than their perceived causes.

#### Risk and awareness

Awareness of the issue of climate change, and perceptions and dimensions of associated risks, were discussed. With regards to public awareness of climate change, many people noted increasing media coverage of the issue. Participants in three groups talked about the media “hyping up” the risks of climate change and being selective with what they report in order to sell news stories. Several participants viewed climate change as a present risk to themselves or their broader community, but most people felt the problem to be far away in space and/or time. All four groups that discussed temporal dimensions of risk had participants that talked about climate change as a long-term threat, for their future and future generations. Several people expressed worry for their children, such as: “If we don’t do something then we are definitely going to be in trouble down the road.” Geographically, some people from all groups recognized climate change as a global risk. However, there was disagreement on whether climate change poses a local risk to southern Manitoba and to participants personally; some said yes (e.g. through drought impacting farmers) and others said no (e.g. their community is far from rising seas and melting ice). As one person expressed: “I think [Brandon’s] pretty distanced, both in terms of time and geography from people who are impacted.” People in two groups expressed not knowing enough about climate change to talk about the risks.

#### Solutions and politics

The scientific basis and political context around climate change and climate solutions frequently came up in discussions. The credibility of climate science and use of data projections to inform solutions was commonly brought up among those more skeptical of climate change. People in four groups expressed beliefs that there is not a long enough historical record of climate data to know whether the current change is part of a natural cycle or is human caused. Some questioned how scientists can project future climates when they cannot even accurately predict the weather several days in advance, without seemingly understanding the differences between climatology and meteorology. Others defended the science, saying they believe in the consensus among experts that climate change is “a real thing.” Funding for climate research was also debated; some were critical of government spending tax dollars on climate change research, while another person criticized governments for silencing scientists speaking the truth about climate change. One participant in Brandon described climate change as “a political hot topic now.”

There was some discussion on what solutions are needed to address climate change. Four groups talked about the need to “change our ways” and take action. Several people talked about adaptation and the need for an energy transition off of fossil fuels. One group raised concerns that potential solutions could be economically driven, which might also lead to negative environmental consequences that are not yet known.

#### Differences between high and low climate concern groups

**Fig. 2:**
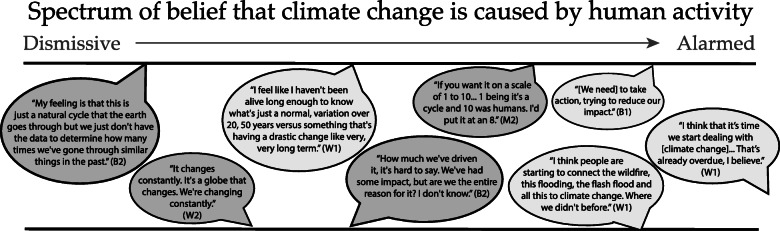
Quotes from focus group participants in high climate concern (W1, B1, M1) and low climate concern (W2, B2, M2) groups, illustrating a spectrum of beliefs of the anthropogenic nature of climate change

Skepticism and misconceptions around climate change were much more prevalent amongst the low concern groups than high concern groups (Table 3). Codes related to climate skepticism appeared more commonly in low concern groups than high concern groups. More people in the low concern groups (particularly B2 and M2) expressed views of climate change as natural, cyclical, and evolving, as compared to the high concern groups. Some participants in B2 and M2 talked about climate change as a “manufactured problem,” created or exaggerated for profit, or through “fear-mongering” by governments or organizations. As one person described: “You know there’s people that make big money off putting the fear of the environment into people and, it could be a product that they’re selling or it could be the taxes or it could be all sorts of things, but there’s money to be made by scaring people.” Skepticism was less common in the high concern groups, but not absent. In B1 for example, some people talked about how the climate has always been changing and will continue to change into the future. In general, there was a spectrum of beliefs regarding to what degree climate change is natural and to what degree it is exacerbated by humans (Fig. 2).

As far as climate impacts, warming temperatures were not talked about in the low concern groups in Winnipeg or Brandon, and there was debate about whether temperatures have changed in Morden. On the other hand, warming temperatures were discussed by participants in all the high concern groups. Some impacts were talked about across high and low concern groups, such as weather extremes and flooding, impacts to health and growing seasons, and melting Arctic ice.

### Public perceptions of Lyme disease

Similar to climate change, there was a wide range of knowledge and perceptions of Lyme disease among participants. Some people had high levels of awareness and knowledge on Lyme disease (particularly those who had known someone with the disease) while others had little to none. All groups had one or more participants who knew someone who had had Lyme disease, including a family member in one case, and two groups had five or more people who reported knowing someone with the disease. Participants often shared stories of these people’s experiences with the disease in the discussions. Four main themes emerged during analyses to describe participants’ perceptions of Lyme disease and findings are presented accordingly: lack of knowledge; risk and awareness; causes of disease spread; and illness representations (Table 4). Notable geographic differences between communities are discussed.

**Table 4:** Lyme disease perceptions themes and sub-themes that emerged through the process of qualitative analysis. The most common 5–6 sub-themes are shown per theme

Lyme disease perceptions			
Lack of Knowledge	Causes of Spread	Risk and Awareness	Illness Representation
• Public • Medical	• Climate change • Habitat change • Migration • Weather • Natural spread, natural cycles of ticks	• Temporal and geographic dimensions of risk • Increasing public awareness • Source of risk information • Diagnosis or risk increasing • Risk is not new • Absence of risk	• Causes • Symptoms • Treatment and prevention • Trajectory • Consequences • Definition

#### Lack of knowledge on Lyme disease

There was expression across all groups of a lack of knowledge on the disease and the need for education among both the public and the medical community. Two people who had recently moved to the province said they had not heard about the disease until arriving in MB. Many people talked about a lack of knowledge among doctors in diagnosing Lyme disease and a lack of preparedness of the healthcare system to respond to the emerging disease. One person described their impression that “it can go on for years and years and years before doctors can figure it out.” Numerous participants in three different groups shared stories of people seeking treatment outside of Canada after not receiving proper diagnosis or treatment at home. Several people also raised questions around why a vaccine has not been developed for Lyme disease.

#### Risk and awareness

In discussions of awareness of the disease and perceptions of the risks associated, all groups agreed that they perceive increasing awareness around Lyme disease now compared to the past. People reported hearing about it most commonly through increasing media coverage, as well as family or friends, the healthcare system, and celebrities.

While there was agreement that awareness of the disease is increasing, there was debate in four groups about whether disease risk/incidence is actually increasing or whether it is a matter of increasing diagnosis and reporting. Several people made statements similar to the sentiment shared by this Brandon participant: “I think we have to be cautious around the increasing diagnosis numbers because medical professionals are more aware of it now... Things that may not have been diagnosed as Lyme disease but actually were Lyme disease, even 10, 15 years ago, are now more likely to be diagnosed as Lyme disease.”

Participants in five groups expressed a lack of feeling of risk or worry about Lyme disease. In four groups, people said “ticks have always been around,” and in three groups people said they have always taken cautionary and preventative behaviours. Fewer participants shared feelings of fear around increasing risk to humans and animals, and beliefs that Lyme disease “can happen to anyone.” There was disagreement in several groups about whether or not Lyme-carrying ticks can be encountered in urban environments such as city parks and lawns.

#### Causes of spread of Lyme disease

The drivers of the spread of blacklegged ticks carrying Lyme disease were also discussed. Most groups talked about ticks increasing and migrating because of warmer temperatures and more hospitable climates. “The habitat is changing so they can come up here, and instead of just visiting and going home, they can start living here,” one person explained. Some also talked about tick spread via bird, animal, or human migration. Two groups talked about humans moving closer to woodland tick habitat due to urban sprawl and therefore having more contact with ticks. Two other groups discussed natural cycles of tick populations making their numbers greater in some years than others, suggesting their increase is not climate-related. Those who were more skeptical of the role of climate change in increasing Lyme disease risk often brought up that there are other invasive species – such as zebra mussels in Lake Winnipeg – and suggested that that non-climatic factors are also involved in species movement. One person summarized the sentiment that ticks just spread on their own by saying: “They got legs, they move or they ride on something that is moving.”

#### Illness representations

In discussions of the disease itself, people talked about the causes, symptoms, consequences, trajectory, definition, treatment, and prevention. Most people identified ticks as the cause Lyme disease spread, and some people in four groups named the blacklegged (deer) tick specifically. Fewer people knew how to identify this tick and where it can be found.

Participants identified a range of symptoms of Lyme disease, including the bullseye rash, fatigue, memory impacts, mental health effects (e.g. depression, anxiety), aching joints, and neurological symptoms. People in five groups talked about the disease being chronic, while four groups understood the disease as potentially fatal and numerous people said they knew someone who had died of Lyme disease. Several people compared the symptoms to other illnesses such as lupus or ringworm. As one person said: “It’s very symptomatic, like lupus from what I understand, which you really can’t tell exactly what you’ve got.”

There was disagreement in several groups about whether Lyme disease can be cured completely or just treated to lessen symptoms. As one Morden participant said, “I’ve heard it’s very hard to diagnose and lots of the tests come back like false negatives. So even if they suspect that, certain testing isn’t always accurate.” Other consequences of the disease that were raised included missing work or losing one’s job, relationship problems, expensive treatment, and having to move to access care.

The feelings people expressed about the disease range from fear or disgust of ticks, fear of Lyme disease, both or neither. One participant expressed their discomfort with ticks: “I’m not panicked about Lyme disease per se … but I just don’t like them. They freak me out at the end of the day.” These feelings were associated with the presence/absence of preventative behaviours. Common preventative behaviours identified include using bug spray, wearing full-length clothes, tucking pants into socks, and doing tick checks after being outside. For some, their fear of ticks or Lyme disease is what motivates them to adopt these behaviours. On the other hand, there were many people who said they have preventative behaviours out of habit, as something they have always done to deal with ticks, not in response to Lyme disease. As one person explained, “I’ve never honestly thought, ‘Oh hey, I’m going to get a tick,’ when I’m tucking, or spraying, or whatever. It’s just this is what you do to go outside.”

#### Geographic differences

Participants in focus groups outside of Winnipeg seemed to have more familiarity with ticks in general, more often reported taking precautions against tick bites, and seemed to be more active on applied health adaptation despite their lower overall climate change concerns. Many had an established disposition towards ticks from previous exposure – because “ticks have always been around” or fear of the ticks since “they’re just gross” – and often did not distinguish between blacklegged ticks and wood ticks. People in all four groups from Brandon and Morden talked about having preventative behaviours from growing up with ticks. Speaking of the precautions they take, one Brandon participant said: “It’s just part of going outside... It’s like you really have to protect yourself from outside.” Someone else added “It was just like common sense.” Several people expressed that these habits were instilled by parents from a young age. One participant talked about tick checks and deterrents growing up on a farm, explaining “that’s what we do, we grew up that way.” All 11 participants in one of the Morden groups reported doing tick checks after being out in the woods, and several people in this group talked about tucking pants into socks or using duct tape on their pant legs when hunting.

**Fig. 3:**
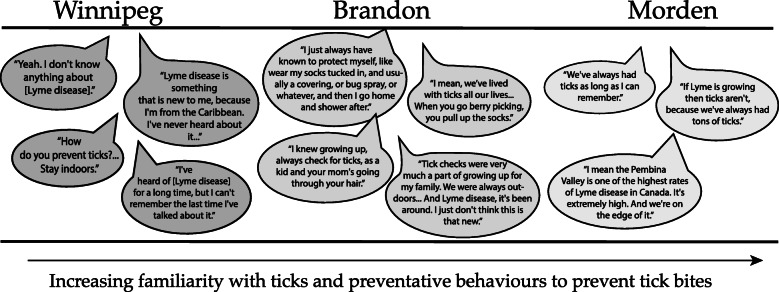
Quotes from focus group participants in the three communities illustrating attitudes and familiarity with ticks and preventative behaviours

Conversely, in Winnipeg people spoke of preventative behaviours that *could* be taken, but fewer people spoke of personal experience or habits of taking these precautions. Also, when asked to name preventative behaviours, people in both Winnipeg groups said “don’t go outside” which did not come up in any of the more rural groups. Participant quotes illustrating these geographic differences are shown in Fig. 3.

## Discussion

Overall, this study aimed to explore public perceptions of climate change and Lyme disease in order to provide a baseline to inform risk communication around the health impacts of climate change, especially in a Canadian Prairie geography prone to climate skepticism. The qualitative approach richly surfaced a nuanced understanding of people’s perceptions of the causes, effects, and responses to climate change and Lyme disease and how this was influenced by their overall levels of climate concern and geographic locations within the province of Manitoba.

### Climate change perceptions

Four key findings emerged in response to the first research objective (see Table 5 for a summary of these findings).

**Table 5:** Summary of the key findings and implications concerning climate change perceptions

Key Findings	Implications:
• A wide range of knowledge on climate change exists, with a general superficial understanding – and in some cases deep misunderstanding – of the issue.	• More science education and communication on climate change is needed in southern MB. • Future research is needed to better understand the relationship between knowledge of climate change, risk perception, and support for action.
• Few people denied climate change outright, but some degree of skepticism was present, mostly (but not exclusively) in the low climate concern groups and those in more rural areas.	• Climate change perspectives are complex, intersectional, and varying in a manner that creates a spectrum of viewpoints. • Climate communications targeting these audiences should take into consideration the extent and drivers of skepticism.
• Perceived uncertainty around climate change was often expressed and linked to a perceived lack of credibility, reliability or consensus in climate science as well as a lack of understanding of science.	• Climate communicators should promote overall scientific literacy, while paying specific attention to the importance of framing messages in an accessible and relatable manner.
• Temporal, social, and geographical dimensions of psychological distancing of climate change also arose in discussions across groups.	• Results suggest that perhaps localizing and personalizing climate change messages is useful – and is supported by the literature – yet further research is needed to understand how psychological distancing might function within the Prairies especially in the context of other potential drivers (e.g. faith, political beliefs, views of nature, etc).

There was a range of knowledge on climate change amongst participants from across southern MB – from those able to cite specific scientific climate models and emissions targets, to those who denied the reality of anthropogenic climate change. Many people seemed focused on issues that are more visible in their daily lives – pollution in waterways, sprawl of urban development, lack of trees planted in their communities – using climate change as a “catch-all” to encompass these issues. While sprawl and smog are not unrelated to climate change, the nuances of those relationships were not articulated. This, combined with the near absence of fossil fuels and greenhouse gas emissions from discussions, reflects a lack of deep understanding of climate change among many participants. In fairness, climate change may remain too abstract and invisible for some participants, and thus seemingly failed to resonate with people in the way that more observable issues such local garbage and pollution do. These results suggest that, given the diversity of backgrounds and contexts of populations in southern MB, enhanced science education within the region may be beneficial to support a greater understanding regarding climate change among the population. This raises an important question: what level of knowledge regarding the mechanisms of anthropogenic climate change and associated perception of risk is necessary for people to support action?

Studies that have sought comprehensive models to explore the relationship between climate change knowledge and risk perception have had mixed results [8] and likely require a more nuanced understanding of risk perceptions and their drivers – including cultural theory of risk, heuristics, trust, social values, social amplification and psychological distancing [5] – which are specific to the Prairies and its people. Indeed, cross-country research that includes Canada often concludes that the national public is “reasonably well informed” [61], which does not take into account the important regional variation that takes place within the Prairie context.

Beliefs on climate change also ranged widely among focus groups. Though fewer people outright denied climate change, numerous were unconvinced or skeptical, similar to findings from other countries [62, 63]. The reasons for skepticism are complex and link with threatened values, ideology, media influence, and deliberate attempts to undermine and call into question climate science [61]. The fact that low climate concern groups had more unconvinced, skeptics, and deniers [64] than the high concern groups suggests that beliefs about climate change are positively correlated with levels of concern. Similar results have been found in previous studies in other countries [65, 66]. In their landmark study on “Global Warming’s Six Americas”, Leiserowitz et al. found that among the six categories of audiences in the American public – alarmed, concerned, cautious, disengaged, doubtful, and dismissive – belief in the reality of global warming and worry about the impacts were highest among the ‘alarmed’ and lowest among the ‘dismissive’ [65]. The results here also indicate more skepticism and denial in rural areas as compared to urban, which aligns with existing data on climate opinions in the region [44]. Humanity’s role in causing climate change was debated in Morden and Brandon groups, where survey data indicates that only about half the population in the regions – 45 and 51% respectively— believe that the earth is warming partly or mostly because of human activity, compared to 60% nationally [44].

Participants’ uncertainty around climate change was often expressed as a perceived lack of credibility, reliability, or consensus in climate science, with an emphasis on the issue being “exaggerated” or the historical climate record being insufficient. The climate psychology and risk literature explore the ways in which perceived uncertainty of climate science is used to justify inaction. Research suggests that uncertainty is one of the main reasons that people will ignore climate change and act in short-term self-interest [64]. This perception of uncertainty may be linked with decades of media framings that have painted a picture of scientific debate around the core tenants of the issue, which “frequently result in illusory, misleading, and counterproductive debates among publics and within and between policy communities” [67] (p 215) [68]. Messaging on climate change has been an issue of deep politicization, lobbying, and controversy whereas public communication on Lyme disease has not been targeted in the same way. Perceptions of uncertainty can also arise from the cautionary and probabilistic language that scientists employ in communicating findings on climate change, using words like ‘likely’ and ‘uncertain’ to talk about degrees of confidence in the data supporting a conclusion [64, 66]. The results here show that for some participants the uncertainty arose from a fundamental lack of understanding of climate science, reflected in statements that conflate weather predictions with climate projections. And perhaps believing the science is uncertain is cognitively easier for people to process than the alternative. Economist and climate psychologist Stoknes’ work on cognitive dissonance surrounding climate change – the tension that arises when people try to reconcile the magnitude of the problem of climate change while recognizing that their actions are contributing to that problem – shows how people cognitively make space for doubt as a coping mechanism [14].

In addition to uncertainty, temporal, social, and geographical dimensions of psychological distancing of climate change [6] were exemplified in the discussions. Whether they were talking about impacts elsewhere in the world or worry for their children’s future, the majority of participants described climate change as being worse in other places or times. Another illustration of distancing in the focus groups was the placing of blame or responsibility for the problem on others, likely indicative of the cognitive dissonance that arises in knowing that one’s actions are contributing to the problem and searching for ways to lessen feelings of guilt [14]. Relatedly, many participants expressed low feelings of personal risk, reflecting what Kahneman and Tversky call the ‘optimism bias’ [69]. A minority of people said they felt like climate change posed a risk to them personally, which aligns with quantitative survey data for the region [44]. While there is extensive literature that suggests that making climate change psychologically “close” has greater potential to engage people and increase willingness for action – by showing that climate impacts are here, now, and impacting people like them [6, 70] – contrasting work suggests that in some cases closeness of perceived impacts can potentially cause people to deny or disengage out of fear [71]. Some argue that more balanced climate communications are required, which both report local impacts to “reduce the distance”, as well as focus on global identity and connectedness to “bridge the distance” [72].

One way that climate change can potentially become psychologically close is through personal experience. Participants in high and low concern groups and across the spectrum of beliefs talked about changes they have experienced such as local flooding. Personal experience of climate change-related events in relation to climate beliefs, risk perceptions, and willingness to act has been extensively studied in recent years [8, 71, 73, 74]. Experiential processes are one of four main factors influencing public risk perception on climate change, among socio-demographic, cognitive, and socio-cultural factors [8, 75]. In a survey of the American public looking at climate beliefs and experiences, Myers et al. found evidence that experiences affect people’s beliefs differently: those who are more engaged on climate change are more likely to seek out evidence supporting their existing beliefs (‘motivated reasoning’) while those less engaged are more likely to be shaped by their personal experiences of impacts (‘experiential learning’) [74, 76]. Others have also suggested that the connection between personal experience and conviction on climate change is not as straightforward as it may seem [64, 77]. Because an individual weather event can never specifically be attributed to climate change, and thus climate change cannot be experienced directly, research has shown that people are more likely to interpret events in the frame of pre-existing prejudices or values or through observations mediated by the media [8, 62, 77]. This seemed to be the case for example in focus group discussions around the unusually early snow storm in southern MB in October 2019, which came up in many of the groups; some participants talked about it as a sign of climate change, while others argued that snow in October was proof that the climate is not warming. There’s a substantial body of evidence that demonstrates recent or remembered weather events – e.g. temperature anomalies like being unseasonably cold or early snow – can affect beliefs and perceptions [61, 74].

### Lyme disease perceptions

With regards to the second research objective, four key findings emerged to describe Lyme disease perceptions (Table 6).

**Table 6:** Summary of the key findings and implications concerning Lyme disease perceptions

Key Findings	Implications
• There was a wide range of experience and concern about Lyme disease, with most participants lacking detailed knowledge on Lyme disease and some sharing misinformation.	• While public health communication efforts have been successful in increasing awareness of Lyme disease, more specific education and ‘myth busting’ information is needed.
• Despite discussions of the disease as serious, the majority of participants are not worried about the risk of Lyme disease.	• Public health communication may consider emphasizing the benefits and ease of adopting preventative behaviours, if risk messaging is not sufficient to motivate behavioural change and associated safety.
• Differences in perceptions emerged according to urban and rural groups, with rural groups having more awareness and preventative behaviours related to ticks generally. In this way, rural people seemed to be leaders in Lyme disease adaptation, and were uncomfortable with climate change being presented as a rationale for health prevention.	• Despite significant research that suggests “coupling” climate and health information to spur action, this exploratory study suggests that with climate skeptical audiences this “coupling” might be counter-productive. Indeed, people may question the importance of health adaptation when it is conflated with their pre-existing doubt and denial of climate change.
• While skepticism arose in some discussion of Lyme disease – particularly concerning the connection between climate change and Lyme disease – there was no denial of the disease.	• The ways in which skepticism arises in discussions of Lyme disease – especially when explicitly linked with climate change – suggests that decoupling the issues in communication materials might be beneficial especially in regions with known skepticism.

While the climate psychology and communications literature around personal experience of climate change-related impacts largely focus on weather events, experience and perceptions of health impacts have received less attention. The increasing incidence of Lyme disease, due in part to climate change, is an interesting, if complex, example to take. The results herein show a range of levels of knowledge and concern about Lyme disease amongst participants, paralleling those on climate change. In general, participants lacked detailed knowledge on Lyme disease, and in some cases shared misinformation they perceived to be true. For instance, people in many groups said the disease was chronic, incurable, and/or fatal, while research says that lasting neurological and cardiac symptoms leading to death are extremely rare [46, 78] and most experts agree that “chronic” Lyme disease does not exist [79]. Despite a lack of knowledge about the disease among many, there was a high incidence of participants reporting to know someone personally who has or had Lyme disease. This is surprising, given that the number of confirmed cases was only 2.4 per 100,000 people in Manitoba as of 2015 [46]. This may be illustrative of under-detection of Lyme disease in Canada [80], or that people suffering with unexplained symptoms look to Lyme disease as an explanation, leading many physicians and experts to call it a “catch-all disease” [79] (p136). The uncertainty and misinformation among participants reflect the complexities and controversies that surround the disease in public discourse and, to a lesser degree, the medical community [79, 81, 82].

Interestingly, despite discussion of the disease as serious and relatively common, the majority of people said they are not worried about the risk of Lyme disease. This could reflect a similar optimism bias as came up in discussions of climate change, and have been recorded in Lyme disease perception research elsewhere [79]. This raises a similar question around the relationship between levels of knowledge, concern, and changes in behaviour.

Differences in perceptions and behaviours around Lyme disease between urban and rural groups offer interesting insight for public health communication. Research from elsewhere in Canada has emphasized the importance of regionally-specific Lyme disease communications, taking into account differences in audience knowledge and characteristics between emerging and endemic risk areas [40, 41]. Results here suggest that communication on Lyme disease should consider the historic presence of other tick species, as well as the epidemiological status of the region and common activities of target audiences. Most participants who grew up and live in more rural areas where other tick species have been present for a long time already had awareness and preventative behaviours that would also apply to preventing Lyme-carrying blacklegged tick bites, pointing to a potential source of adaptation and resilience. At the same time, the fact that their familiarity with ticks in general made some people unconcerned about blacklegged ticks points to a potential vulnerability in responding to the specific emerging risk of Lyme disease. Thus communication approaches for these populations should focus more on distinguishing different species of ticks, reinforcing adaptive behaviours, dispelling common misinformation, and illustrating the unique disease risks brought by the blacklegged tick. This also supports the idea that a different communication approach is needed for southern Manitoba than would be in parts of Canada where other species of ticks such as the wood tick have not been common historically and therefore the arrival of the blacklegged tick is more alarming.

### The relationship between climate change and Lyme disease perceptions

Overall, participants’ perceptions of climate change and Lyme disease varied along similar spectrums from denial, skepticism, unconvinced/uncertain, recognition of low risk, and recognition of high risk. However, the results do not indicate that climate change and Lyme disease perceptions were necessarily linked, which creates an opportunity to explore the different ways that people perceive these issues and the relevant frames with which to communicate with them. Lyme disease perceptions did not seem to differ notably between low and high climate concern groups. Climate skeptical participants were sometimes highly concerned about the risk of Lyme disease, for example.

**Fig. 4:**
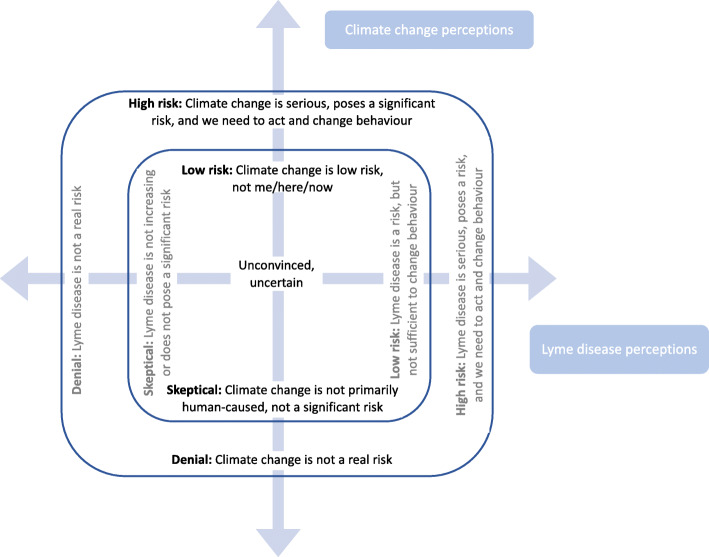
A proposed model for the relationship between climate change and Lyme disease risk perception, which is designed to support targeted interventions that allows for audience segmentation and appropriate communication framing

In light of these findings, it is clear that parallels emerged in participants’ perceptions of climate change and Lyme disease with regards to the spectra of belief and risk perception, ranging from denial of the problem to belief that it poses a serious risk. Since climate change risk perception did not necessarily indicate Lyme disease risk perception – and vice versa – the relationship between the two risks is not necessarily straight forward, which suggests that risk communication will need to understand and take into account where the target audience is on both axes of risk. Inspired by seminal risk perception theory (e.g. [83]), a conceptual model was developed exploring the relationship between perceptions of climate change and Lyme disease, which may elucidate the various perspectives and potential strategies for targeted risk communication with these audiences (Fig. 4).

In Fig. 4, people could fall anywhere along the axes of climate change and Lyme disease perceptions. For example, someone who sees climate change as a high risk and does not feel that Lyme disease is a risk (perhaps because they live in an urban centre and have not had much exposure to ticks or tick-borne diseases) would fall in the upper left quadrant. Someone who is a climate skeptic but views Lyme disease as a serious threat – even if they do not believe that the spread of Lyme disease is affected by climate change – would land in the bottom right quadrant. While all positions on the figure are theoretically possible, there were no people in these groups who fully denied Lyme disease.

This exploratory model suggests that public risk communication regarding health and climate change should be framed in ways that account for both the climate opinions and health perceptions of the audience. We suggest that tailored language and framing is required for different audiences, which foregrounds the issue that will resonate people’s pre-existing concerns the most. For audiences that are highly skeptical of climate change, communication may be most effective if health and climate messages are “strategically decoupled,” foregrounding public health risks and preventative behaviours rather than climate science which may lead the audience to reject the subsequent health information. In no way are we suggesting that information should be concealed from an audience, rather we are suggesting that it is important that risk communicators understand the interplay and associated implications when using health and climate messaging together, especially in areas with known climate skepticism. Depending on one’s objectives, our study and exploratory model may provide guidance on how to work with certain audiences, and when it may be appropriate to link or “strategically decouple” health and climate risk communications.

While this exploratory model is focused on Lyme disease and climate change *risk perceptions*, it must be noted that the relationship between the two issues from climatological and epidemiological perspectives is complicated. There are other factors in addition to climate change that influence the range expansion and population dynamics of blacklegged tick vectors, host species, and the bacteria that cause Lyme disease, and thus it is hard to exclusively isolate the role of climate change. This may make space for climate skeptics to point to other explanations for the rise of Lyme disease. Future studies should explore perceptions around other climate-affected diseases and health outcomes to see if perhaps they are more closely linked on different issues. The implications and mechanisms for effective public health framing in climate communications should also be further investigated and, ideally, it’s possible to understand how enhanced climate education reduces climate skepticism and the associated need for “strategic decoupling.”

### Limitations and opportunities

The present study has some limitations due to the nature of the focus group methods employed; for example, it is possible that some participants’ views were swayed by others or that some voices dominated the discussion. Future research should use methods of independent participation such as interviews and surveys to confirm the exploratory conclusions drawn here. Ongoing research might also increase sample sizes within the region in order to further test their interrelationships and association with salient and potentially causal factors such as urban and rural residency, high and low levels of climate change concern, and Lyme disease experience that emerged in the present study. Additionally, the present study did not account for participants’ ethnocultural background; since culture greatly influences worldview, future studies should explore how identity affects these types of perceptions. Despite these limitations, the study was effective and generated some important conclusions.

Importantly, the relationships in our proposed theoretical framework are based on the exploratory data and should be tested in subsequent quantitative studies to further investigate and substantiate it as a robust tool for audience segmentation and associated communications. Future studies could compare populations by geography, community size, or sociodemographic characteristics to see which perception profiles are more common and how their response to communication materials differ. Despite being a preliminary model, we believe this represents an important opportunity within the Canadian Prairies – and perhaps other areas with known climate skepticism – to engage a diversity of audiences in conversation and messaging regarding applied climate and health adaptation regardless of one’s underlying worldviews and associated climate literacy.

## Conclusions

While climate change stands to impact every aspect of our society, it has been suggested that a focus on health implications in climate communications can maximize engagement from a wider audience [11, 13, 14]. In order to design public health communication strategies that increase the salience of climate change and health, regional risk perceptions of climate change and health impacts must be better understood. This focus group study was conducted in three communities in southern Manitoba to explore public perceptions of climate change and climate-affected tick-borne Lyme disease, filling a gap since there is a lack of this type of research in the Canadian Prairies. The results indicate a broad range of knowledge and perceptions of risk on both climate change and Lyme disease, reflective of the controversies that exist in the public discourse around both issues. Personal experiences play a role in shaping perceptions on these issues, and may exemplify experiential learning or conversely may be invoked to justify or support existing views and attitudes.

With respect to climate change perceptions, many participants were found to have a relatively low understanding of the mechanism of climate change, namely human activities producing greenhouse gas emissions, and a range of uncertainty or skepticism around the issue. With regards to Lyme disease perceptions, the results suggest a potential “double-edged sword” of resilience and vulnerability: the long-term presence of wood ticks in the region have led many to adopt preventative behaviours that could also protect against Lyme disease, while at the same time these existing tick species have led some to have lower levels of concern about the emergence of Lyme-carrying blacklegged ticks. Given the spectrum of knowledge, attitudes, and risk perceptions on climate change and Lyme disease, the task of bringing the two issues together to inform risk communication materials tailored to engage different audiences and motivate action is complex.

Better understanding the implications of climate skepticism, particularly in rural areas across the Canadian Prairies, is critical for ongoing public health communications on climate impacts, and more work in this area is necessary. An important and somewhat surprising implication of the study is the fact that risks associated with climate and risks associated with certain health issues are not necessarily linked in the public’s minds, which much of the literature conversely suggests are elegantly aligned to advance risk perception and adaptive action [12–16]. However, in this study, our exploration of aligning climate and health issues may have, in some cases, contributed to confusion, doubt, and further cynicism especially amongst an already (higher than the national average) climate skeptical public.

Interestingly, some people who lived in rural areas and expressed doubts about climate change, were highly knowledgeable about health adaptation practices that help reduce the transmission of climate-affected Lyme disease. That rural people were leaders regarding Lyme disease prevention demonstrates the important contribution that skeptical audiences can make towards health adaptation regardless of their underlying climate beliefs. This suggests that while climate education continues to infuse society, we do not necessarily have to wait for its full uptake to support effective risk communication regarding Lyme disease and other climate-linked infectious diseases. In certain contexts, employing communication frames that strategically decouple climate and health communications might be more effective to achieve applied adaptation to attendant risks. That climate messaging might adversely affect health messaging – in climate skeptical contexts – and ultimately create maladaptation is a very serious issue that must be avoided. Given very few studies have been conducted within the Canadian Prairie context, this paper offers new insights regarding how to engage individuals and communities within this geography that may be beneficial for other jurisdictions facing similar climate skepticism.

Our exploratory conceptual model of climate and Lyme risk perceptions may help orient researchers working within a climate skeptical context, and provide navigation for when and where to strategically decouple messaging. This model offers an opportunity for more nuanced audience segmentation, which might generate more targeted framing to maximize effective risk communication and greater uptake of applied adaptation options. At the same time, while this decoupling approach may increase adoption of preventative health behaviours among certain skeptical audiences, it must be evaluated against the benefits of increasing public awareness of the links between climate and health more generally. Climate education is paramount and, ideally, the need for strategic decoupling wanes as larger societal awareness regarding the need for climate action and adaptation grows, especially within contexts that currently trend towards doubt and denial. Indeed, finding climate communication approaches that honour the diverse worldviews of the public is critical as we seek to prepare both urban and rural communities in the face of ever-increasing climate change and associated health impacts like vector-borne Lyme disease.

## Data Availability

The data that support the findings of this study are available from The University of Winnipeg but restrictions apply to the availability of these data, which were used under license for the current study, and so are not publicly available.

## Appendix A

### Focus Group Questionnaire

Risk Perception of climate change and Lyme disease

*To start off, I want us to brainstorm a list. When I say the phrase “climate change” - what comes to mind? It can be anything …*

- *Do you see climate change as a risk to you personally?*
- *How big is this risk?*
- *How about to your community? In what way?*
- *How big is this risk to your community?*

*OK, how about Lyme disease - what are some of the first things that come to mind when I say Lyme disease?*

- *When did you first hear about Lyme disease? Have you seen anything or heard anything about it lately? Where was that?*
- *Do you know anyone with Lyme disease?*
- *Do you think there is a risk of Lyme disease in Manitoba? How big is this risk? Is it getting bigger or smaller?*
- *How do you prevent Lyme disease? What are the things you’re supposed to do?*
- *Do you think Lyme disease is connected to climate change? In what way?*

Communication materials presentation

*I’ve got some materials here I want to show you and get your thoughts on. We’ll go one-by-one.* (Present in randomized order).

Article - Discussion

*I’ve got an article here for you to read on your own. While you’re reading it, I want you to do two things:*

1. *With the green highlighter, I want you to highlight everything that’s new to you, stuff you didn’t know - green is “news to you.”*
2. *You’ve each got one dot. At the end, when you’re done reading, put that dot next to the most surprising thing, the thing that had the most impact on you.*

- *What’s the key message of this article, if you had to put it into words?*
- *Did this seem trustworthy or credible? Why/why not?*
- *Was it clear? Or confusing? What parts?*
- *How did your understanding of Lyme disease change - if at all?*
- *Do you think you’d change your behaviour because of this article? In what way?*
- *Where would you expect to see this article? What’s the best way to get it in front of you? Would you actually read it? What would motivate you to read it?*
- *Is there anything the university could do to improve this article?*

Video - Discussion

*Now, I’ve got a short video to show you. We’ll just watch it once and then we’ll chat about it …*

- *What’s the key message of this video, if you had to put it into words?*
- *What was one thing you learned you didn’t know?*
- *Did you connect with the video emotionally? If so, how?*
- *How did your understanding of Lyme disease change - if at all?*
- *Do you think you’d change your behaviour? In what way?*
- *Is there anything the university could do to improve this video?*

Maps - Discussion

*Now, I want to show you some map(s). You can get up and study these a little closer if you want. I’m going to give you a couple of minutes to really have a look at these …*

- *What’s your key takeaway from these maps?*
- *Did they seem credible or not? Why/why not?*
- *Were they clear? Or confusing? What parts?*
- *Did your understanding of Lyme disease change? If so, how?*
- *Do you think you’d change your behaviour because of these maps? In what way?*
- *Where would you expect to see these maps?*
- *Is there anything the university could do to improve these maps?*

Comparison of Materials and Closing

- *Thinking across the three options, did the different approaches to communication impact you differently?*
- *Do you see value in each approach? If so/if not, why?*
- *Any final thoughts? If you would prefer to write any thoughts or reflections you have you can do so on the paper provided and leave them on your tables for us to collect afterwards.*

## Appendix B

### Coding scheme and definitions

**Table:**

Perceptions of climate change			
Level 1 code	Level 2 code	Level 3 code	Definition
**Climate Impacts**			
	Temperature changes		Abnormality in temperature ranges or rate of change compared to previous
	Weather changes, extremes		
		Floods, Rain, Storms	Floods, typhoons, changes in precipitation, hurricanes.
		Snow	Abnormal snowfall, snowstorms and blizzards.
		Weather changes	Extreme weather fluctuations, changes in weather throughout the years or abnormal weather.
	Health and human impacts		Direct or indirect climate-related health impacts on humans (e.g. increase in asthma, diseases) and other impacts on humans such as through food production (e.g. food shortages)
	Ice, oceans, water		Impacts of climate change on water or oceans (e.g. rising sea levels, glaciers and ice melting, pollution of oceans).
	Loss of wildlife, habitat, biodiversity		Extinction or decline of animals and/or habitat
	Pests, invasive species		More pests and invasive species becoming more apparent because of climate change and changing weather (e.g. ticks, pine beetles, etc.)
	Fire, drought		Increasing wildfires, drought, dryness.
	Air quality, smog		Smog, worsened air quality, air pollution in cities, etc.
	Skepticism of impacts and changes (S)		Doubts or denial of impacts being climate-driven
**Causes**			
	Culture/mentality		Attitudes and culture of society as a whole being the problem for climate change (e.g. culture of dependency)
	Economy		Climate change being driven by economic profit
	Fossil fuels, emissions		Fossil fuels, vehicles, other emissions sources that contribute to climate change.
	Government inaction		Government failing to act or impeding action on climate change
	Pollution		Solid waste (e.g. garbage) and air pollution (worsened air quality) as part of the problem of climate change.
	Population growth and urban sprawl		Growth of cities and town transforming and impeding on natural environments, contributing to climate change
	Responsibility for the problem		The role of humans and specific cities or countries in driving the problem of climate change
	Manufactured problem (S)		Denial of climate change as a real problem, belief that it is manufactured for profits or political gain, or a perception that climate change is being blown out of proportion
**Risk & Awareness**			
	Lack of public knowledge		A deficit of public knowledge on climate change or the risks in general.
	Media coverage		
		Hearing it from media	Getting information on climate change through the media
		Media hype (S)	Belief that climate change is being exaggerated by the media
	Change is constant (S)		Belief that climate change is natural or normal, or part of a cycle that is constantly changing
	Skepticism or denial of the risk (S)		Belief that climate change is not a risk personally or more generally, that the problem is not worsening
	Temporal risk		When people believe the risk of climate change will be realized
	Geographic risk		
		Globally	The risk of climate change for people around the world, or specifically in other countries
		Locally	The risk of climate change for the specific area or city that participants live in
		Personally	The risk of climate change to participants personally
**Solutions and politics**			
	Adaptation		The necessity and measures of adapting to climate change.
	Energy transition		The necessity and measures of transitioning away from fossil fuel energy.
	Individual and collective action		The role of individual and/or collective action
	Disbelief or skepticism of solutions (S)		Skepticism or doubt around some of the climate change solutions (e.g. electric cars)
	Climate science		Scientific consensus on climate change
	Lack of scientific knowledge (S)		Belief that there is a lack of climate or weather data, and other scientific information on climate change
	Government, politics		The political dimensions of climate change
**Perceptions of Lyme Disease**			
**Level 1 code**	**Level 2 code**	**Level 3 code**	**Definition**
**Lack of Knowledge**			
	Public		A deficit of knowledge on Lyme disease amongst the public, such as lack of public education on symptoms, consequences, and causes.
	Medical		A deficit of knowledge on Lyme Disease amongst doctors and medical professionals, such as misinformation or lack of capacity around diagnosis.
**Causes of Spread**			
	Climate Change		Blacklegged ticks spreading and migrating because of specifically climate change.
	Habitat change		Blacklegged ticks migrating or moving because of loss of habitat cause by humans (e.g. deforestation, encroachment)
	Migration		Blacklegged ticks moving around on animals or otherwise naturally migrating to new areas
	Weather		Tick spreading is due to weather or humidity.
	Spread is not climate change related (LS)		The cause of ticks spreading is specifically not climate change related
	Natural spread, cycles in ticks (LS)		Spread of ticks to new areas is natural or part of a pattern or cycle in their populations
**Illness Representation**			
	Causes		The source of Lyme Disease, such as the specific ticks that carry the disease
	Symptoms		The physical (e.g. bullseye rashes) and mental (e.g. depression) impacts of Lyme Disease.
	Definition		Participants feelings about ticks (e.g. disgust) or feelings about Lyme Disease (e.g. scared).
	Consequences		Outcomes of having Lyme Disease (e.g. loss of job, needing to travel to get treatment, etc.)
	Trajectory		The course of living with Lyme Disease (e.g. curable, chronic)
	Treatment & Prevention		
		Preventative Behaviour	What can be or has been done to avoid getting bitten by ticks and contracting Lyme Disease (e.g. applying bug spray)
		Treatment	The process, location, cost, etc. of medical treatment to cure or lessen Lyme disease symptoms
**Risk and Awareness**			
	Temporal and geographic dimensions of risk		Who could get Lyme Disease, where they could get it (e.g. walking their dog, in their grass, etc.) and when they could get it (e.g. seasonality, etc.)
	Increasing public awareness		Hearing about Lyme Disease more, increasing discussion of the disease in the public sphere
	Source of risk information		Sources of information about Lyme Disease (e.g. radio, news, relationships)
		Media coverage	Coverage of Lyme Disease information through media specifically
	Diagnosis or risk increasing (LS)		Skepticism amongst participants about whether the risk of Lyme Disease is increasing or if increasing number of reported cases is just a result of doctors becoming more aware of Lyme Disease and how to diagnose it.
	Risk is not new (LS)		Ticks are not new, and have been around for a long time.
	Absence of risk (LS)		Lack of concern about Lyme Disease, belief that the disease risk is not significant

## Abbreviations

MB: Manitoba
CC: Climate change
W1: Winnipeg Focus Group 1
W2: Winnipeg Focus Group 2
B1: Brandon Focus Group 1
B2: Brandon Focus Group 2
M1: Morden-Winkler Focus Group 1
M2: Morden-Winkler Focus Group 2

## References

[1] Costello A, Abbas M, Allen A, Ball S, Bell S, Bellamy R, Friel S, Groce N, Johnson A, Kett M, Lee M, Levy C, Maslin M, McCoy D, McGuire B, Montgomery H, Napier D, Pagel C, Patel J, de Oliveira JAP, Redclift N, Rees H, Rogger D, Scott J, Stephenson J, Twigg J, Wolff J, Patterson C (2009). Managing the health effects of climate change. Lancet and University College London Institute for Global Health Commission. Lancet..

[2] Canadian Public Health Association (2017). Lancet Countdown 2017: Briefing for Canadian Policy Makers.

[3] Watts N, Amann M, Ayeb-Karlsson S, Belesova K, Bouley T, Boykoff M, Byass P, Cai W, Campbell-Lendrum D, Chambers J, Cox PM, Daly M, Dasandi N, Davies M, Depledge M, Depoux A, Dominguez-Salas P, Drummond P, Ekins P, Flahault A, Frumkin H, Georgeson L, Ghanei M, Grace D, Graham H, Grojsman R, Haines A, Hamilton I, Hartinger S, Johnson A, Kelman I, Kiesewetter G, Kniveton D, Liang L, Lott M, Lowe R, Mace G, Odhiambo Sewe M, Maslin M, Mikhaylov S, Milner J, Latifi AM, Moradi-Lakeh M, Morrissey K, Murray K, Neville T, Nilsson M, Oreszczyn T, Owfi F, Pencheon D, Pye S, Rabbaniha M, Robinson E, Rocklöv J, Schütte S, Shumake-Guillemot J, Steinbach R, Tabatabaei M, Wheeler N, Wilkinson P, Gong P, Montgomery H, Costello A (2017). The lancet countdown on health and climate change: from 25 years of inaction to a global transformation for public health. Lancet..

[4] Fischhoff B (2012). Risk analysis and human behaviour.

[5] Hagen B (2016). Public perception of climate change: policy and communication.

[6] Spence A, Poortinga W, Pidgeon N (2012). The psychological distance of climate change. Risk Anal.

[7] Sterman JD (2011). Communicating climate change risks in a skeptical world. Clim Chang.

[8] van der Linden S (2015). The social-psychological determinants of climate change risk perceptions: towards a comprehensive model. J Environ Psychol.

[9] Leiserowitz A (2006). Climate change risk perception and policy preferences: the role of affect, imagery, and values. Clim Chang.

[10] Lieske DJ, Wade T, Roness LA (2014). Climate change awareness and strategies for communicating the risk of coastal flooding: a Canadian maritime case example. Estuar Coast Shelf Sci.

[11] Nisbet MC (2009). The Ethics of Framing Science. Communicating Biological Sciences: Ethical and Metaphorical Dimensions.

[12] Maibach EW, Kreslake JM, Roser-Renouf C, Rosenthal S, Feinberg G, Leiserowitz AA (2015). Do americans understand that global warming is harmful to human health? Evidence from a national survey. Ann Glob Health.

[13] Maibach EW, Roser-Renouf C, Leiserowitz A (2008). Communication and marketing as climate change-intervention assets. A public health perspective. Am J Prev Med.

[14] Stoknes PE (2015). What we think about when we try not to think about global warming: toward a new psychology of climate action.

[15] Myers TA, Nisbet MC, Maibach EW, Leiserowitz AA (2012). A public health frame arouses hopeful emotions about climate change: a letter. Clim Chang.

[16] Weathers MR, Kendall BE (2016). Developments in the framing of climate change as a public health issue in US newspapers. Environ Commun.

[17] Sol Hart P, Nisbet EC (2012). Boomerang effects in science communication: how motivated reasoning and identity cues amplify opinion polarization about climate mitigation policies. Commun Res.

[18] Weathers MR (2013). Newspaper coverage of global warming and climate change (GWCC) as a public health issue. Appl Environ Educ Commun.

[19] Depoux A, Hémono M, Puig-Malet S, Pédron R, Flahault A (2017). Communicating climate change and health in the media. Public Health Rev.

[20] Harrison S, Macmillan A, Rudd C (2020). Framing climate change and health: New Zealand’s online news media. Health Promot Int.

[21] Nisbet MC, Price S, Pascual-Ferra P, Maibach E, Maibach EW. Communicating the public health relevance of climate change: a news agenda building analysis. Working Paper, American University. 2010. Accessed from: https://www.researchgate.net/publication/242451772.

[22] Akerlof KL, Delamater PL, Boules CR, Upperman CR, Mitchell CS (2015). Vulnerable populations perceive their health as at risk from climate change. Int J Environ Res Public Health.

[23] Kabir MI, Rahman MB, Smith W, Lusha MAF, Azim S, Milton AH (2016). Knowledge and perception about climate change and human health: findings from a baseline survey among vulnerable communities in Bangladesh. BMC Public Health.

[24] Hathaway J, Maibach EW (2018). Health implications of climate change: a review of the literature about the perception of the public and health professionals. Curr Environ Health Reports.

[25] Li J, Xu X, Ding G, Zhao Y, Zhao R, Xue F (2016). A cross-sectional study of heatwave-related knowledge, attitude, and practice among the public in the Licheng District of Jinan City, China. Int J Environ Res Public Health.

[26] Akerlof K, Debono R, Berry P, Leiserowitz A, Roser-Renouf C, Clarke KL (2010). Public perceptions of climate change as a human health risk: surveys of the United States, Canada and Malta. Int J Environ Res Public Health.

[27] Cardwell FS, Elliott SJ (2013). Making the links: Do we connect climate change with health? A qualitative case study from Canada. BMC Public Health.

[28] Berry P, Clarke K-L, Pajot M, Hutton D, Ford JD, Berrang-Ford L (2011). Risk perception, health communication, and adaptation to the health impacts of climate change in Canada. Climate change adaptation in developed nations: from theory to practice.

[29] Ogden NH, Lindsay LR, Morshed M, Sockett PN, Artsob H (2009). The emergence of Lyme disease in Canada. Cmaj..

[30] Bouchard C, Dibernardo A, Koffi J, Wood H, Leighton P, Lindsay L (2019). Increased risk of tick-borne diseases with climate and environmental changes. Can Commun Dis Rep.

[31] Ogden NH, Radojević M, Wu X, Duvvuri VR, Leighton PA, Wu J (2014). Estimated effects of projected climate change on the basic reproductive number of the Lyme disease vector ixodes scapularis. Environ Health Perspect.

[32] Nelder M, Wijayasri S, Russell C, Johnson K, Marchand-Austin A, Cronin K (2018). The continued rise of Lyme disease in Ontario, Canada: 2017. Can Commun Dis Rep.

[33] Lieske DJ, Lloyd VK (2018). Combining public participatory surveillance and occupancy modelling to predict the distributional response of Ixodes scapularis to climate change. Ticks Tick Borne Dis.

[34] Ogden NH, St.-Onge L, Barker IK, Brazeau S, Bigras-Poulin M, Charron DF (2008). Risk maps for range expansion of the Lyme disease vector, Ixodes scapularis, in Canada now and with climate change. Int J Health Geogr.

[35] Ogden NH, Koffi JK, Pelcat Y, Lindsay LR (2014). Environmental risk from Lyme disease in central and eastern Canada: a summary of recent surveillance information. Can Commun Dis Rep.

[36] Gabriele-Rivet V, Koffi JK, Pelcat Y, Arsenault J, Cheng A, Lindsay LR, Lysyk TJ, Rochon K, Ogden NH (2017). A risk model for the Lyme disease vector ixodes scapularis (Acari: Ixodidae) in the prairie provinces of Canada. J Med Entomol.

[37] Bouchard C, Aenishaenslin C, Rees EE, Koffi JK, Pelcat Y, Ripoche M, et al. Integrated social-behavioral and ecological risk maps to prioritize local public health responses to Lyme disease. Env Health Persp. 2018;126(4):1–14. 10.1289/EHP1943 PMC6071748 29671475

[38] Chilton NB, Curry PS, Lindsay LR, Rochon K, Lysyk TJ, Dergousoff SJ (2020). Passive and active surveillance for Ixodes scapularis (Acari: Ixodidae) in Saskatchewan, Canada. J Med Entomol.

[39] Ripoche M, Lindsay LR, Ludwig A, Ogden NH, Thivierge K, Leighton PA (2018). Multi-Scale Clustering of Lyme Disease Risk at the Expanding Leading Edge of the Range of Ixodes scapularis in Canada. Int J Environ Res Public Health.

[40] Aenishaenslin C, Ravel A, Michel P, Gern L, Milord F, Waaub J (2014). From Lyme disease emergence to endemicity : a cross sectional comparative study of risk perceptions in different populations. BMC Public Health.

[41] Aenishaenslin C, Bouchard C, Koffi JK, Pelcat Y, Ogden NH (2016). Evidence of rapid changes in Lyme disease awareness in Canada. Ticks Tick Borne Dis.

[42] Aenishaenslin C, Bouchard C, Koffi JK, Ogden NH (2017). Exposure and preventive behaviours toward ticks and Lyme disease in Canada: results from a first national survey. Ticks Tick Borne Dis.

[43] Crang K (2010). Knowledge and perception of Lyme disease in Manitoba: implications for risk assessment.

[44] Mildenberger M, Howe PD, Lachapelle E, Stokes LC, Marlon JR, Gravelle T (2016). The distribution of climate change public opinion in Canada*. PLoS One.

[45] He W (2012). An evaluation of prairie producer attitudes towards climate change.

[46] Gasmi S, Ogden NH, Lindsay LR, Burns S, Fleming S, Badcock J, Hanan S, Gaulin C, Leblanc MA, Russell C, Nelder M, Hobbs L, Graham-Derham S, Lachance L, Scott AN, Galanis E, Koffi JK (2017). Surveillance for Lyme disease in Canada: 2009-2015. Can Commun Dis Rep.

[47] Leighton PA, Koffi JK, Pelcat Y, Lindsay LR, Ogden NH (2012). Predicting the speed of tick invasion: an empirical model of range expansion for the Lyme disease vector Ixodes scapularis in Canada. J Appl Ecol.

[48] Manitoba G of. Tick-borne Diseases https://www.gov.mb.ca/health/publichealth/cdc/tickborne/index.html. Accessed 26 May 2020.

[49] Creswell JW (2014). A concise introduction to mixed methods research.

[50] Peters K (2017). Your human geography dissertation: designing, doing, delivering.

[51] Olausson U (2011). “We’re the ones to blame”: citizens’ representations of climate change and the role of the media. Environ Commun.

[52] Jovchelovitch S (2001). Social representations, public life, and social construction. Representations of the social: Bridging theoretical traditions.

[53] Blaikie N (2010). Designing social research.

[54] Wibeck V (2014). Social representations of climate change in Swedish lay focus groups: local or distant, gradual or catastrophic?. Public Underst Sci.

[55] Zuckerman-Parker M, Gary S (2008). The town hall focus group: a new format for qualitative research methods. Qual Rep.

[56] Krueger RA (2000). Casey MA. Focus groups: a practical guide for applied research, 3rd ed.

[57] Morgan DL (1997). The focus group guidebook; focus group kit.

[58] Baxter J (2009). Content analysis.

[59] Hay I (2010). Qualitative research methods in human geography.

[60] Hannah DR, Lautsch BA (2011). Counting in qualitative research: why to conduct it, when to avoid it, and when to closet it. J Manag Inq.

[61] Whitmarsh L, Capstick S (2018). Perceptions of climate change. Psychology and Climate Change: Human Perceptions, Impacts, and Responses.

[62] Whitmarsh L (2011). Scepticism and uncertainty about climate change: dimensions, determinants and change over time. Glob Environ Chang.

[63] Upham P, Whitmarsh L, Poortinga W, Purdam K, Darnton A, Mclachlan C (2009). Public attitudes to environmental change: a selective review of theory and practice-executive summary.

[64] Marshall G (2015). Don’t even think about it : why our brains are wired to ignore climate change.

[65] Leiserowitz A a, Maibach E, Roser-renouf C (2009). Global Warming’s “Six Americas.”.

[66] Visschers VHM (2018). Public perception of uncertainties within climate change science. Risk Anal.

[67] Boykoff MT, Smith J, Lever-Tracy C (2015). Media presentations of climate change. Routledge handbook of climate change and society.

[68] Boykoff MT, Boykoff JM (2007). Climate change and journalistic norms: a case-study of US mass-media coverage. Geoforum..

[69] Kahneman D, Tversky A (1982). Intuitive prediction: biases and corrective procedures. Judgment under Uncertainty.

[70] Akerlof K, Maibach EW, Fitzgerald D, Cedeno AY, Neuman A (2013). Do people “personally experience” global warming, and if so how, and does it matter?. Glob Environ Chang.

[71] McDonald RI, Chai HY, Newell BR (2015). Personal experience and the “psychological distance” of climate change: an integrative review. J Environ Psychol.

[72] Loy LS, Spence A (2020). Reducing, and bridging, the psychological distance of climate change. J Environ Psychol.

[73] Hornsey MJ, Fielding KS, Mcstay R, Reser JP, Bradley GL, Greenaway KH (2015). Evidence for motivated control: understanding the paradoxical link between threat and efficacy beliefs about climate change. J Environ Psychol.

[74] Myers TA, Maibach EW, Roser-Renouf C, Akerlof K, Leiserowitz AA (2013). The relationship between personal experience and belief in the reality of global warming. Nat Clim Chang.

[75] Corcoran PB, Helgeson J, van der Linden S, Chabay I, Wals A, Corcoran P (2012). The role of knowledge, learning and mental models in public perceptions of climate change related risks. Learning for sustainability in times of accelerating change.

[76] Weber EU (2013). Psychology: seeing is believing. Nat Clim Chang.

[77] Whitmarsh L (2008). Are flood victims more concerned about climate change than other people? The role of direct experience in risk perception and behavioural response. J Risk Res.

[78] Stanek G, Strle F (2018). Lyme borreliosis-from tick bite to diagnosis and treatment. FEMS Microbiol Rev.

[79] Peretti-Watel P, Ward J, Lutaud R, Seror V (2019). Lyme disease: insight from social sciences. Med Mal Infect.

[80] Lloyd V, Hawkins R (2018). Under-detection of Lyme disease in Canada. Healthcare..

[81] Aguero-Rosenfeld ME, Wormser GP (2015). Lyme disease: diagnostic issues and controversies. Expert Rev Mol Diagn.

[82] van Hout MC (2018). The controversies, challenges and complexities of Lyme disease: a narrative review. J Pharm Pharm Sci.

[83] Slovic P (1987). Perception of Risk. Science.

